# Device-measured physical activity and type 2 diabetes mellitus risk

**DOI:** 10.3389/fendo.2023.1275182

**Published:** 2023-12-18

**Authors:** Wenzhao Li, Weizhi Zhang, Zhenhua Xing

**Affiliations:** ^1^ Department of Orthopedics, The Second Xiangya Hospital of Central South University, Changsha, Hunan, China; ^2^ Department of Cardiovascular Surgery, The Second Xiangya Hospital of Central South University, Changsha, Hunan, China; ^3^ Department of Emergency Medicine, Second Xiangya Hospital, Central South University, Changsha, Hunan, China; ^4^ Emergency Medicine and Difficult Diseases Institute, Central South University, Changsha, Hunan, China

**Keywords:** type 2 diabetes mellites, physical activity, light physical activity, moderate physical activity, vigorous physical activity

## Abstract

**Objectives:**

We investigated how device-measured physical activity (PA) volume (PA energy expenditure [PAEE]) and intensity (fraction of PAEE from moderate-to-vigorous PA [FMVPAEE]) were associated with the incidence of type 2 diabetes mellites (T2DM).

**Methods:**

This population-based prospective cohort study included 90,044 participants. The primary exposures were PAEE and FMVPAEE. The secondary exposures were energy expenditure exerted during light, moderate, and vigorous PA and their fraction of PAEE.

**Results:**

Each 1-SD increase in PAEE was associated with a 17% lower risk of T2DM (hazard ratio [HR]: 0.83, 95% confidence interval [CI]: 0.78–0.98). Each 1-SD increase in FMVPAEE was associated with a 21% lower incidence of T2DM (HR: 0.79, 95% CI: 0.74–0.83). Achieving the same PA volume (KJ/kg/day) through vigorous PA (HR: 0.88, 95% CI: 0.85–0.91) was more effective in preventing T2DM than moderate PA (HR: 0.97, 95% CI: 0.96–0.98) and light PA (HR: 0.99, 95% CI: 0.98–1.00).

**Conclusion:**

A higher PA volume is associated with a lower incidence of T2DM. Achieving the same PA volumes through higher-intensity PA is more effective than low-intensity PA in reducing T2DM incidence.

## Introduction

Type 2 diabetes mellitus (T2DM) has become a major public health problem and its incidence continues to increase rapidly worldwide (1, 2). One potential modifiable factor for preventing T2DM is physical activity (PA) (3). Meta-analysis of prospective studies has demonstrated that PA is associated with a lower incidence of T2DM. However, most of the studies on this topic have used self-report tools (e.g., questionnaires) to measure PA, which are less accurate than device-measured energy expenditure (EE), and thus, potentially leading to bias and obscuring the actual nature and magnitude of the association between PA and the incidence of T2DM (4, 5). Recently, an increasing number of people have used wearable accelerometers to measure their PA. These wearable devices have the advantage of objectively measuring PA volume, intensity, duration, and distance traveled (6, 7) and could, therefore, provide insights into PA and incidence of T2DM.

The guidelines on PA recommend that adults undertake moderate-to-vigorous PA (MVPA) equivalent to, or more intense than, steady walking (8, 9). For example, the World Health Organization recommends that adults undertake at least 150–300 min of moderate PA (MPA) or 75–150 min of vigorous PA (VPA) weekly. This recommendation is based on evidence that an equivalent amount of time spent in MVPA confers greater health benefits than that of light PA (LPA) (10, 11). Strain et al. (6) found that MVPA was associated with lower incidence of all-cause mortality than that with LPA. However, whether MVPA contributes to a lower incidence of T2DM remains unclear. Recent guidelines recognize that LPA, the main contributor to PAEE, is beneficial for health (12, 13), but do not specify whether PA volume (intensity × time, evaluated by PA EE [PAEE]) or PA intensity alone is more important to health. Furthermore, these guidelines do not specify how to maximize the health benefits of PA (LPA for a longer time compared with MVPA for a shorter time). Individualized strategies for PA may be more appealing and more likely to be accepted by different individuals.

The UK Biobank, which includes 103,686 participants with valid accelerometer data, is the largest study of accelerometer-measured PA to date. In this study, we used the data from the UK Biobank to assess the dose-response association between PA (volume and intensity) and the incidence of T2DM. Furthermore, we compared the magnitude of LPA, MPA, and VPA for the prevention of T2DM to explore how individuals could maximize the health benefits of PA through individualized PA strategies.

## Methods

### Study design and population

The Biobank study enrolled more than 500,000 participants aged 40–69 years across the UK between 2006 and 2010. The design of the Biobank study has been described previously (14). This cohort study was approved by the North West Multi-Center Research Ethics Committee, and all participants provided informed consent. All participants received questionnaire, physical examination, and laboratory tests. More information about the Biobank cohort can be found at www.ukbiobank.ac.uk.

Between 2013 and 2015, PA data were collected from 103,686 UK Biobank participants with Axivity AX3 wrist-worn triaxial accelerometers (**Supplementary Figure 1**). Participants wore an accelerometer on their dominant wrist for 7 days at all times, including while working, walking, and sleeping. Further details about data collection and processing can be found in previous reports (6, 7). Participants with diabetes (n=4,456) before PA measurement were excluded from this study. Participants with implausibly high activity values (average vector magnitude of >200 mg) were also excluded. A total of 9,186 participants were excluded owing to problems with PA data. Overall, 90,044 participants were included in the analysis (**Supplementary Figure 2**).

### PA volume and intensity

PAEE (KJ/kg/day), which was approximated using simple wearable accelerometers such as the Apple watch, was used as the measure of PA volume per day. We transformed the raw data into PAEE with precision using the validated doubly labeled water method (15, 16). PAEE per day was estimated using the quadratic equations from White et al. (16), who converted with high accuracy the average wrist acceleration into PA-related EE (6, 16). LPA, MPA, and VPA was determined by wrist acceleration magnitude in 30–125 mg, 125–400 mg, and >400 mg. EE from LPA (LPAEE), MPA (MPAEE), and VPA (VPAEE) was estimated as the sum of the LPAEE, MPAEE, and VPAEE (**Supplementary Table 1**). The fraction of PAEE from MVPAEE (FMVPAEE), which was estimated as the sum of EE from vector magnitude >125 mg divided by total PAEE, was used to assess the PA intensity. The fraction of PAEE from LPA, MPA, and VPA (FLPAEE, FMPAEE, and FVPAEE, respectively) was also estimated using a similar method and were expressed as a percentage. The primary exposures in this study were PAEE and FMVPAEE. The secondary exposures were LPAEE, MPAEE, VPAEE, FLPAEE, FMPAEE, and FVPAEE.

### Definition of T2DM

The diagnosis of T2DM (ICD10 code I48) was ascertained using diagnostic codes linked to hospital visits and death records. Participants with incident T2DM were identified through the “first occurrence of health outcomes” defined by a three-character International Statistical Classification of Diseases and Related Health Problems 10th Revision code (field ID 131351). The follow-up period of each participant was measured from the date of finishing PA measurement to the date of developing T2DM, death, or censoring (May 31, 2022, field ID: 131350), whichever occurred first.

### Covariates

Age, sex, race, Townsend deprivation index (TDI), smoking (never/former/or current) and alcohol consumption (times per week), dietary score, employment status (with/without), mobility limitation (yes/no), history of myocardial infarction, diabetes, and cholesterol-lowering medication at recruitment were identified using questionnaires, interviews, and medical records (**Supplementary Figure 1**). The TDI was calculated based on specific areas of output from previous national censuses that allowed identification of the most and least deprived areas of the country, and provided information on the issues faced by people living in different parts of the country (17). Dietary scores (ranging from 0 to 4) were calculated based on the following dietary factors: cooked or raw vegetable intake ≥4 tablespoons/day; fresh fruit intake ≥ three pieces/day; fish intake ≥ twice/week, and processed meat intake ≤ twice/week. Systolic blood pressure (SBP), diastolic blood pressure (DBP), waist circumference (WC), and weight were measured by registered nurses at the assessment center. Body mass index (BMI) was categorized into non-obese (<30 kg/m^2^) and obese (≥30 kg/m^2^).

### Statistical analysis

The baseline characteristics of the included participants are presented as numbers, proportions, or mean ± standard deviation (SD). Continuous variables were compared using Student’s t-test or the Mann–Whitney U test according to the distribution type; and categorical variables were compared using chi-square tests. Missing data were coded as missing indicator categories for categorical variables, such as race, and mean values for continuous variables, such as BMI and WC. Three Cox proportional hazards regression models were used to assess the relationship between PA volume or intensity (continuous or categorical variables) and T2DM risk. Model 1 was unadjusted. Model 2 was adjusted for age, sex, race, WC, BMI, smoking status, alcohol intake frequency, dietary score, employment status, TDI, and mobility limitations. Model 3 was further adjusted for SBP, DBP, cholesterol-lowering medication use, and history of myocardial infarction. Additionally, we used restricted cubic splines with four knots positioned at the 5th, 35th, 65th, and 95th percentiles, to flexibly ascertain the association between PA volume or intensity and the risk of T2DM. This analysis was conducted after adjusting for Model 3 variables. Sensitivity analysis was performed to verify the robustness of the results. First, interaction and stratified analyses were conducted according to age (<60 and ≥60 years), sex, race, BMI categories (non-obese, <30 kg/m^2^; obese, ≥30 kg/m^2^) and employment status (yes or no). Second, to avoid bias in the Cox proportional hazards analysis, we used competing risk models to estimate the risk of T2DM. Competing events of all-cause mortality as a permanent condition may prevent the occurrence of T2DM. Third, we excluded participants with missing data avoid bias caused by missing data. All statistical analyses were 2-sided, and P-values <0.05 were considered statistically significant. All analyses were performed using Stata 17 (StataCorp, College Station, TX, USA).

## Results

### Baseline characteristics of included participants

Of the 90,044 included participants who provided valid accelerometer data, 57% were female with a mean age of 56 ± 7.8 years. During the median follow-up period of 7.3 years (interquartile range [IQR], 6.8–7.8 years), 1,791 participants developed T2DM (2.7 per 1000 person-years). The mean PAEE was 46.5 ± 13.8 KJ/kg/day, and the mean FMVPAEE was 30.8 ± 9.2%. Participant characteristics according to quartiles of PAEE are shown in **Table 1**. Participants who had more PAEE were younger, more likely to be female, employed, less likely to have mobility limitations, and be taking cholesterol-lowering medication. Additionally, they had lower DBP, SBP, WC, and BMI values. Participants with higher FMVPAEE had similar characteristics to those of participants with higher PAEE (**Supplementary Table 2**).

**Table 1:** Characteristics of included participants based on quartiles of physical activity energy expenditure.

	Quartiles of PAEE				
	Q1	Q2	Q3	Q4	P-value
N	23625	23626	23626	23625	
**PAEE(KJ/kg/day)**	30.35 ± 5.16	40.79 ± 2.28	48.97 ± 2.60	64.29 ± 10.98	<0.01
**FMVPAEE (%)**	21.98 ± 7.09	28.26 ± 6.19	32.94 ± 5.96	40.05 ± 6.43	<0.01
**Age(years)**	58.88 ± 7.27	56.93 ± 7.62	55.44 ± 7.71	53.38 ± 7.63	<0.01
**Sex, female (%)**	11796 (49.93%)	13388 (56.67%)	13987 (59.20%)	14034 (59.40%)	<0.01
**Race, White (%)**	21717 (91.92%)	21508 (91.04%)	21404 (90.60%)	21129 (89.43%)	<0.01
**Smoking status**					<0.01
** Never**	12484 (52.84%)	13447 (56.92%)	13778 (58.32%)	14138 (59.84%)	
** Current**	8975 (37.99%)	8532 (36.11%)	8312 (35.18%)	8078 (34.19%)	
** Quit**	2096 (8.87%)	1582 (6.70%)	1473 (6.23%)	1353 (5.73%)	
Missing	70 (0.29%)	65 (0.27%)	63 (0.26%)	56 (0.24%)	
**Diet**ary **score**					<0.01
** 0**	1174 (4.97%)	901 (3.81%)	941 (3.98%)	837 (3.54%)	
** 1**	5786 (24.49%)	5270 (22.31%)	5023 (21.26%)	4843 (20.50%)	
** 2**	8927 (37.79%)	8831 (37.38%)	8761 (37.08%)	8564 (36.25%)	
** 3**	5811 (24.60%)	6432 (27.22%)	6559 (27.76%)	6791 (28.74%)	
** 4**	1927 (8.16%)	2192 (9.28%)	2342 (9.91%)	2590 (10.96%)	
**Alcohol consumption** (frequency)					<0.01
** Daily**	5227 (22.14%)	5558 (23.55%)	5470 (23.17%)	5326 (22.57%)	
** 3-4/week**	5415 (22.94%)	6091 (25.81%)	6376 (27.00%)	6657 (28.21%)	
** 1-2/week**	5708 (24.18%)	5973 (25.31%)	5962 (25.25%)	6051 (25.64%)	
** 1-3/month**	2855 (12.09%)	2509 (10.63%)	2540 (10.76%)	2367 (10.03%)	
** Occasionally**	2764 (11.71%)	2198 (9.31%)	2044 (8.66%)	1984 (8.41%)	
**Never**	1637 (6.93%)	1274 (5.40%)	1221 (5.17%)	1217 (5.16%)	
**Mobility limitation (%)**	1043 (4.41%)	387 (1.64%)	282 (1.19%)	185 (0.78%)	<0.01
**In employment (%)**	11831 (50.08%)	14047 (59.46%)	15453 (65.41%)	16945 (71.72%)	
**WC (cm^2^)**	93.82 ± 13.71	89.16 ± 12.60	86.77 ± 12.17	83.65 ± 11.52	<0.01
**BMI (kg/m^2^)**	28.46 ± 5.13	26.96 ± 4.41	26.26 ± 4.11	25.17 ± 3.70	<0.01
** Normal weight (<25 kg/m^2^)**	6002 (25.41%)	8502 (35.99%)	9956 (42.14%)	12634 (53.48%)	
** Overweight (25–29.9 kg/m^2^)**	10157 (42.99%)	10288 (43.55%)	9967 (42.19%)	8642 (36.58%)	
** Obese (≥30 kg/m^2^)**	7466 (31.60%)	4836 (20.47%)	3703 (15.67%)	2349 (9.94%)	
**TDI**	−1.58 ± 2.90	−1.78 ± 2.79	−1.80 ± 2.77	−1.74 ± 2.80	<0.01
**DBP (mmHg)**	83.11 ± 10.30	82.05 ± 10.24	81.29 ± 10.22	80.26 ± 10.13	<0.01
**SBP (mmHg)**	141.75 ± 18.89	139.31 ± 18.58	137.77 ± 18.64	135.69 ± 18.21	<0.01
**Total cholesterol (mmol/L)**	5.66 ± 1.16	5.76 ± 1.09	5.76 ± 1.05	5.72 ± 1.02	<0.01
**Cholesterol-lowering medication use**	1431 (6.4%)	1134 (5.0%)	826 (3.7%)	561 (2.5%)	<0.01

BMI, body mass index; DBP, diastolic blood pressure; FMVPAEE, fraction of moderate or vigorous physical activity energy expenditure; PAEE, physical activity energy expenditure; SBP, systolic blood pressure; TDI, Townsend indicator of deprivation; WC, waist circumference.

### Assocation between PA and T2DM risk by intensity of PA

**Figure 1:**
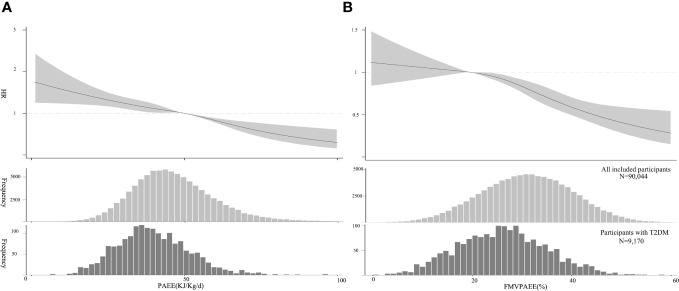
Association between PA and T2DM. **(A)** PAEE (volume) and T2DM, The HRs were estimated using Model 3. **(B)** FMVPA (intensity) and T2DM, HR was estimated by adjusting for Model 3 and PAEE. The HRs are indicated by solid lines and 95% CIs by shaded areas. Distribution of PAEE and FMVPA of the sample and participants with T2DM are shown as histograms, respectively. CI, confidence interval; FMVPA, fraction of PAEE from MVPAEE; HR, hazard ratio; PA, physical activity PAEE, PA energy expenditure; T2DM, type 2 diabetes mellitus.

Participants with incident T2DM had lower PAEE and FMVPAEE than the participants overall (**Figure 1**). When considering the associations of PAEE alone (adjusted for Model 3 without FMVPAEE), each 1-SD increase in PAEE was associated with a 17% decrease in the incidence of T2DM (hazard ratio [HR]:0.83, 95% confidence interval [CI] 0.78–0.98, Model 3, **Supplementary Table 3**). Compared with participants in the first quartile of PAEE, participants in the fourth quartile had a 37% lower incidence of T2DM (HR: 0.63, 95% CI: 0.54–0.73, Model 3, **Supplementary Table 3**). As the PAEE increased, the incidence of T2DM decreased linearly (P<0.01) (**Figure 1A**). Similarly, VPAEE, MPAEE, and LPAEE were associated with a lower incidence of T2DM (**Supplementary Tables 4–6**). However, LAPEE was associated with a higher incidence of T2DM after further adjusting for PAEE (**Supplementary Table 6**).

**Figure 2:**
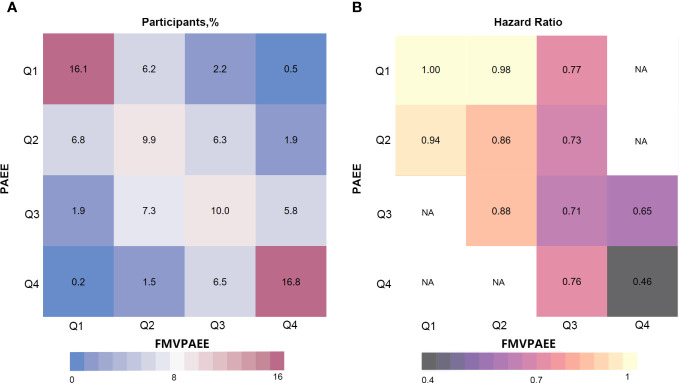
Risk matrix for the joint association between PAEE, FMVPA and T2DM. PAEE and FMVPAEE levels were divided into quartiles. The Q1 group was used as a reference. Hazard ratios were calculated using Cox regression Model 3. NA indicates that there was not enough participants to estimate the hazard ratio. FMVPAEE, fraction of PAEE from MVPAEE; PAEE, PA energy expenditure; T2DM, type 2 diabetes mellitus.

FMVPAEE was associated with a lower incidence of T2DM. Each 1-SD increase in FMVPAEE was associated with a 21% decrease in the incidence of T2DM (HR: 0.79, 95% CI: 0.74–0.83, Model 3, **Supplementary Table 7**). Compared with participants in the first quartile of FMVPAEE, those in the fourth quartile had a 48% lower incidence of T2DM (HR: 0.52, 95% CI: 0.44–0.62, Model 3, **Supplementary Table 7**). After further adjusting for PAEE, the association remained consistent (HR: 0.81, 95% CI: 0.74–0.87, **Supplementary Table 7**). The association was nonlinear, showing flat when the FMVPAEE value was <20%, and becoming steeper when the FMVPAEE value was ≥20% (**Figure 2B**). Similarly, FMPAEE and FVPAEE were associated with a lower incidence of T2DM (**Supplementary Tables 8–9**); however, FLPAEE was associated with a higher incidence of T2DM (**Supplementary Table 10**).

**Figure 3:**
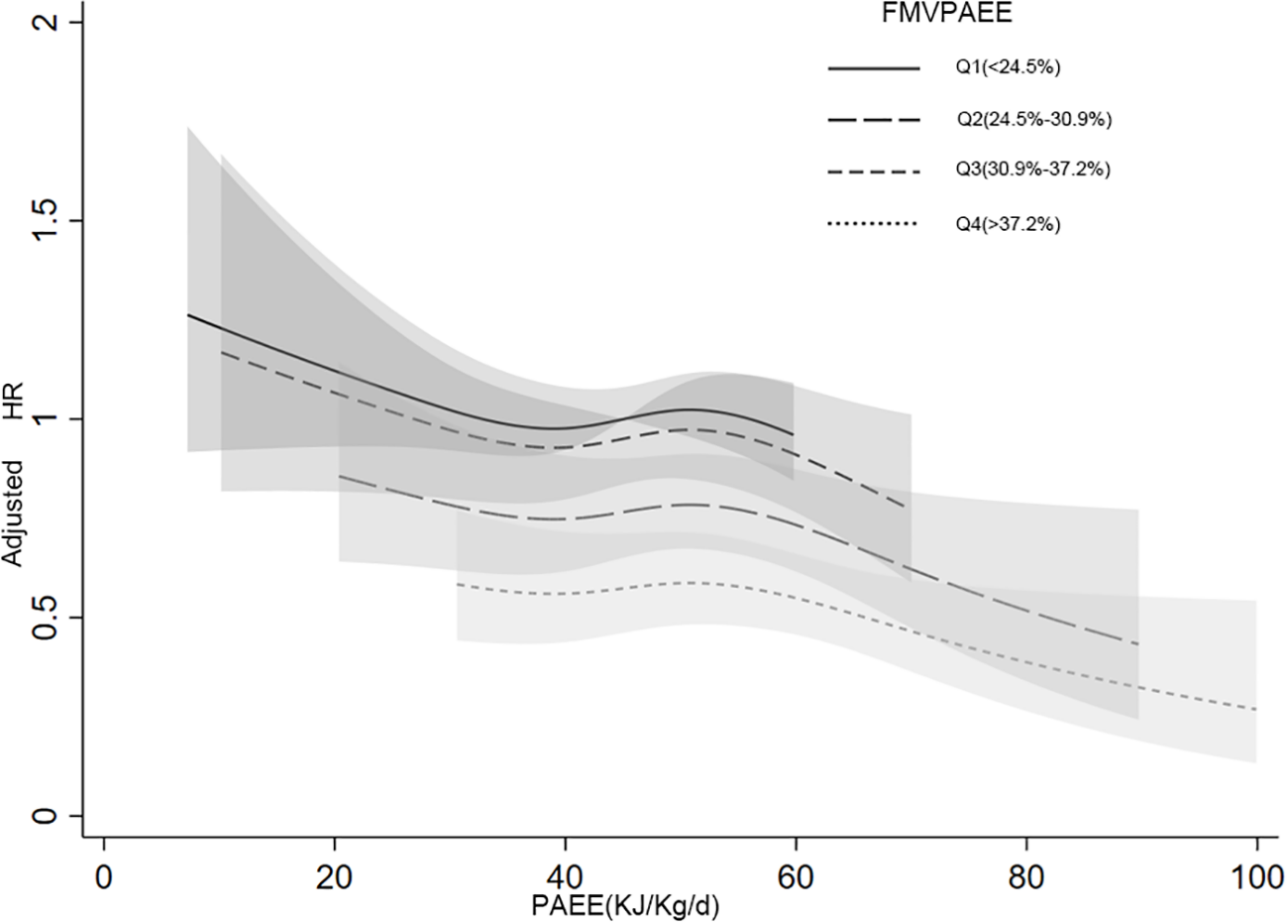
Joint associations between PAEE, FMVPAEE and T2DM by smooth curve. The FMVPA was divided into quartiles. The association between PAEE and T2DM risk was presented in the different FMVPAEE groups. The first quartiles of FMVPAEE and PAEE at 45 KJ/kg/day were used as references. FMVPAEE, fraction of PAEE from MVPAEE; PAEE, PA energy expenditure; T2DM, type 2 diabetes mellitus.

In the interaction analyses of PAEE and FMVPAEE, higher levels of both PAEE and FMVPAEE were associated with a lower incidence of T2DM (**Supplementary Table 11** and **Figure 2**). Participants with higher quartiles of PAEE had a lower incidence of T2DM than participants with the same FMVPAEE. Similarly, Participants with higher quartiles of FMVPAEE had a lower incidence of T2DM than participants with the same quartiles of PAEE. Participants with both PAEE and FMVPAEE in the fourth quartile had a 54% lower incidence of T2DM than those in the first quartile with both PAEE and FMVPAEE (**Supplementary Table 11** and **Figure 2**). In each quartile of FMVPAEE, an increase in PAEE decreases the risk for T2DM accordingly. Achieving the same PAEE, participants with a higher quartile of FMVPAEE had a lower incidence of T2DM (**Figure 3**).

A 1 KJ/kg/day increase in LPAEE was associated with a 1% lower incidence of T2DM (HR: 0.99, 95% CI: 0.98–1.00, Model 3, **Table 2**). MPAEE was associated with a greater protective effect than LPAEE that each 1 KJ/kg/day increase in MPAEE was associated with a 3% lower incidence of T2DM (HR: 0.97, 95% CI: 0.96–98; Model 3, **Table 2**). VPE exerted the most benefit on health that each 1 KJ/kg/day increase in VPAEE was associated with a 12% lower incidence of T2DM (HR: 0.88, 95% CI: 0.85–0.91, Model 3, **Table 2**).

**Table 2:** Magnitude of the associations of light, moderate, and vigorous physical activity energy expenditure and type 2 diabetes mellitus.

	Model 1 HR (95% CI)	Model 2 HR (95% CI)	Model 3 HR (95% CI)
**LPAEE**	0.94 (0.93–0.94)	0.99 (0.98–0.99)	0.99 (0.98–1.00)
**MPAEE**	0.91 (0.90–0.92)	0.97 (0.96–0.97)	0.97 (0.96–0.98)
**VPAEE**	0.76 (0.73–0.79)	0.88 (0.85–0.91)	0.88 (0.85–0.91)

Model 1: Unadjusted.

Model 2: Adjusted for age, sex, race, waist circumference, BMI, smoking status, alcohol intake frequency, dietary score, employment status, Townsend deprivation index, and mobility limitation.

Model 3: Adjusted for the variables in Model 2 and SBP, DBP, cholesterol-lowering medication use, and history of myocardial infarction.

BMI, body mass index; CI, confidence interval; DBP, diastolic blood pressure; HR, hazard ratio; LPAEE, light physical activity energy expenditure; MPAEE, moderate physical activity energy expenditure; SBP, systolic blood pressure; TDI, Townsend indicator of deprivation; VPAEE, vigorous physical activity energy expenditure.

### Sensitivity analysis

The associations of PAEE and FMVPAEE with T2DM were not significantly different when the data were analyzed by age group (age ≥60 years, age <60 years), sex (female or male), race (White, Non-White), smoking (never, current), and employment status (with or without work) (**Supplementary Figure 3**). The magnitude and level of significance of the associations between PAEE or FMVPAEE and T2DM were similar when we used the competing risk models instead of Cox proportional hazards regression and restricted the analysis to participants without missing data (data not shown).

## Discussion

This study was based on the largest study of device-measured PA, which showed that both higher PA volume and intensity were associated with a lower incidence of T2DM. PA intensity plays a remarkably important role in the prospective association between PA and incidence of T2DM over and above PA volume. Higher PA intensity was associated with a lower incidence of T2DM over and above PA volume, and vice versa. This finding is important for individuals who want to undertake individualized PA strategies to maximize the health benefits of PA.

Previous meta-analysis of 78 studies (18,276 T2DM incidence and 104,908 participants) found that higher PA volume measured using self-reported PA (questionnaires) was associated with a reduced risk for T2DM. However, previous studies have shown that self-reported PA has poor agreement with PA quantified using an accelerometer (4, 5, 18, 19). Self-reported time spent on MVPA exceeded the time measured with wearable accelerometers. Previous studies also used daily step counts as a measure of PA,and Daily step counts are easy to understand and can serve as a motivational tool for increasing daily activity (20). However, when a more detailed analysis of PA is required, especially in a research or clinical setting, accelerometers provide a more accurate and comprehensive picture of PA levels. One strength of this study is that we have assessed the health benefits of PA using a more precise method using an accelerometer. Furthermore, the association between PA volume and T2DM risk was linear, without a threshold effect, indicating more PA volume and less risk for T2DM. Previous studies are mainly based on MVPA, ignoring the role of LPA, and thus, have contributed to the nonlinear association between PA volume and T2DM risk (3, 4).

Analysis of both PA volume and intensity showed that all intensities of PA are associated with a lower incidence of T2DM relative to the overall contribution of PA volume. We further compared the magnitudes of LPA, MPA, and VPA with the risk for T2DM and found that VPA was the most effective in reducing T2DM risk, followed by MPA. This finding has clinical implications as personalized strategies for PA may be more appealing and preferred by different individuals.

This study has several strengths. First, we used PA volume and intensity in naturally continuous distributions (instead of comparisons of accumulation pattern groups) using the data from the largest study of accelerometer-measured PA to date, which provided high statistical power to identify individual-level variations between PA volume and intensity. Second, we transformed the accelerometer measurement to a more easily interpretable scale of PAEE with high accuracy using equations that were validated using the reference standard of doubly labeled water (15, 16). This transformation contributes to personalized T2DM risk prediction from wearable accelerometers (e.g., Apple watch) and helps individuals receive immediate feedback on T2DM risk. Third, we adjusted for covariates including BMI, WC, and cholesterol-lowering medication use, which could be mediators or confounders. Finally, our study considered the competing events of deaths as a permanent condition, which may prevent the occurrence of T2DM. These sensitivity analyses showed consistent results, and thus, increasing the confidence of our findings.

Our study has some limitations. First, this was an observational study; therefore, there may be some residual confounding. However, we believe that residual confounding is a minor issue in this study, as we adjusted for multiple key variables associated with the incidence of T2DM. Furthermore, the adjustments had minimal effect on effect size. Second, the intrinsic correlation between PA volume and intensity indicates that joint results cannot be simply interpreted as independent effects, although our analytical methods and large sample size ensured that our results were not biased by collinearity. Third, all the participants were from the UK, indicating that these results may not be conclusive across other ethnicities (e.g., Asians) with different characteristics and lifestyles. Lastly, physical activity data and baseline characteristics were not collected simultaneously. Physical activity data were collected between 2013 and 2015, with a median of 5.7 years of follow up. However, a previous study found that baseline characteristics were generally unchanged in study participants with more than one visit (6).

## Conclusions

Higher PA volumes are associated with a lower incidence of T2DM. For the equivalent PA volume higher-intensity PA is more effective than lower-intensity PA in reducing the incidence of T2DM.

## Data availability statement

The original contributions presented in the study are included in the article/**Supplementary Material**. Further inquiries can be directed to the corresponding author.

## Ethics statement

The studies involving humans were approved by the North West Multi-Center Research Ethics Committee, and all participants provided informed consent. The studies were conducted in accordance with the local legislation and institutional requirements. Written informed consent for participation was not required from the participants or the participants’ legal guardians/next of kin in accordance with the national legislation and institutional requirements.

## Author contributions

WL: Writing – review & editing, Conceptualization. WZ: Writing – original draft. ZX: Writing – original draft, Writing – review & editing.
