# Supplementary material for: Device-measured physical activity and type 2 diabetes mellitus risk
Source: Front Endocrinol (Lausanne). 2023 Dec 18;14:1275182. doi: 10.3389/fendo.2023.1275182 (PMC10764276; doi:10.3389/fendo.2023.1275182)

Supplementary Table 1, calculation of FMVPAEE, FLPAEE, FMPAEE, and FVPAEE

Supplementary Table 2, Baseline Characteristics of included participants based on quantiles of FMVPE

Supplementary Table 3, Associations between PAEE and T2DM

Supplementary Table 4, Associations Between VPAEE and risk of T2DM

Supplementary Table 5, Associations Between MPAEE and risk of T2DM

Supplementary Table 6, Associations Between LPAEE and risk of T2DM

Supplementary Table 7, Associations between FMVPAEE and T2DM

Supplementary Table 8, Associations Between FVPAEE and risk of T2DM

Supplementary Table 9, Associations Between FMPAEE and risk of T2DM

Supplementary Table 10, Associations Between FLPAEE and risk of T2DM

Supplementary Table 11, Associations between joint distribution of PAEE and FMVPAEE and T2DM

Supplementary Figure 1, Timeline of recruitment, data collection and follow-up for included participants

Supplementary Figure 2, Flow diagram of participants in the study

Supplementary Figure 3, Subgroup analysis of the association between PAEE and FMVPAEE and T2DM

**Supplementary Table 1, calculation of FMVPAEE, FLPAEE, FMPAEE, and FVPAEE**

| **PA intensity** | **Category of wrist acceleration (mg)** | **Midpoint of category** | **Activity energy expenditure^a^ (kJ/kg/hour)** | **Average hours**  **per day spent in category^b^** | **Average energy expenditure in kJ/kg/day ^b,c^** |
| --- | --- | --- | --- | --- | --- |
| **Light** | 0-1 | 0.5 | 0.00 | 4.46 | 0.00 |
|  | 1-2 | 1.5 | 0.00 | 2.77 | 0.00 |
|  | 2-3 | 2.5 | 0.00 | 2.13 | 0.00 |
|  | 3-4 | 3.5 | 0.04 | 1.61 | 0.08 |
|  | 4-5 | 4.5 | 0.13 | 1.18 | 0.21 |
|  | 5-6 | 5.5 | 0.22 | 0.84 | 0.26 |
|  | 6-7 | 6.5 | 0.30 | 0.60 | 0.25 |
|  | 7-8 | 7.5 | 0.39 | 0.45 | 0.23 |
|  | 8-9 | 8.5 | 0.47 | 0.35 | 0.21 |
|  | 9-10 | 9.5 | 0.55 | 0.29 | 0.19 |
|  | 10-11 | 10.5 | 0.63 | 0.25 | 0.18 |
|  | 11-12 | 11.5 | 0.71 | 0.22 | 0.18 |
|  | 12-13 | 12.5 | 0.79 | 0.20 | 0.17 |
|  | 13-14 | 13.5 | 0.87 | 0.19 | 0.17 |
|  | 14-15 | 14.5 | 0.94 | 0.18 | 0.18 |
|  | 15-16 | 15.5 | 1.02 | 0.17 | 0.18 |
|  | 16-17 | 16.5 | 1.10 | 0.17 | 0.19 |
|  | 17-18 | 17.5 | 1.17 | 0.16 | 0.20 |
|  | 18-19 | 18.5 | 1.24 | 0.16 | 0.20 |
|  | 19-20 | 19.5 | 1.32 | 0.15 | 0.21 |
|  | 20-25 | 22.5 | 1.54 | 0.72 | 0.25 |
|  | 25-30 | 27.5 | 1.90 | 0.64 | 1.37 |
|  | 30-35 | 32.5 | 2.25 | 0.56 | 1.42 |
|  | 35-40 | 37.5 | 2.59 | 0.49 | 1.42 |
|  | 40-45 | 42.5 | 2.93 | 0.43 | 1.41 |
|  | 45-50 | 47.5 | 3.26 | 0.39 | 1.37 |
|  | 50-55 | 52.5 | 3.59 | 0.35 | 1.36 |
|  | 55-60 | 57.5 | 3.91 | 0.32 | 1.37 |
|  | 60-65 | 62.5 | 4.23 | 0.29 | 1.31 |
|  | 65-70 | 67.5 | 4.55 | 0.27 | 1.32 |
|  | 70-75 | 72.5 | 4.87 | 0.25 | 1.27 |
|  | 75-80 | 77.5 | 5.18 | 0.22 | 1.24 |
|  | 80-85 | 82.5 | 5.49 | 0.20 | 1.21 |
|  | 85-90 | 87.5 | 5.80 | 0.19 | 1.16 |
|  | 90-95 | 92.5 | 6.10 | 0.17 | 1.10 |
|  | 95-100 | 97.5 | 6.40 | 0.16 | 1.09 |
|  | 100-125 | 112.5 | 7.30 | 0.60 | 1.10 |
| **Moderate** | 125-150 | 137.5 | 8.74 | 0.39 | 5.07 |
|  | 150-175 | 162.5 | 10.14 | 0.26 | 3.85 |
|  | 175-200 | 187.5 | 11.49 | 0.17 | 2.87 |
|  | 200-225 | 212.5 | 12.80 | 0.11 | 2.05 |
|  | 225-250 | 237.5 | 14.07 | 0.07 | 1.41 |
|  | 250-275 | 262.5 | 15.30 | 0.05 | 1.07 |
|  | 275-300 | 287.5 | 16.50 | 0.03 | 0.83 |
|  | 300-325 | 312.5 | 17.65 | 0.03 | 0.53 |
|  | 325-350 | 337.5 | 18.77 | 0.02 | 0.56 |
|  | 350-375 | 362.5 | 19.85 | 0.02 | 0.40 |
|  | 375-400 | 387.5 | 20.90 | 0.01 | 0.42 |
| **Vigorous** | 400-425 | 412.5 | 21.91 | 0.01 | 0.22 |
|  | 425-450 | 437.5 | 22.89 | 0.01 | 0.23 |
|  | 450-475 | 462.5 | 23.83 | 0.01 | 0.24 |
|  | 475-500 | 487.5 | 24.74 | 0.01 | 0.25 |
|  | 500-600 | 550 | 26.85 | 0.02 | 0.27 |
|  | 600-700 | 650 | 29.79 | 0.01 | 0.60 |
|  | 700-800 | 750 | 32.19 | 0.01 | 0.32 |
|  | 800-900 | 850 | 34.05 | 0.00 | 0.34 |
|  | 900-1000 | 950 | 35.38 | 0.00 | 0.00 |
|  | 1000-1100 | 1050 | 36.18 | 0.00 | 0.00 |
|  | 1100-1200 | 1150 | 36.45 | 0.00 | 0.00 |
|  | 1200-1300 | 1250 | 36.45 | 0.00 | 0.00 |
|  | 1300-1400 | 1350 | 36.45 | 0.00 | 0.00 |
|  | 1400-1500 | 1450 | 36.45 | 0.00 | 0.00 |
|  | 1500-1600 | 1550 | 36.45 | 0.00 | 0.00 |
|  | 1600-1700 | 1650 | 36.45 | 0.00 | 0.00 |
|  | 1700-1800 | 1750 | 36.45 | 0.00 | 0.00 |
|  | 1800-1900 | 1850 | 36.45 | 0.00 | 0.00 |
|  | 1900-2000 | 1950 | 36.45 | 0.00 | 0.00 |
|  | ≥2000 | 2050 | 36.45 | 0.00 | 0.00 |
| **Total** |  |  |  | 24.00 | 45.57 |

1. Using the quadratic equation from dominant wrist vector magnitude into activity-related energy expenditure measured in J/min/kg : -10.58 + 1.1176*(1.5 + .8517*x) + 2.9418*sqrt((1.5 + .8517*x)) - 0.00059277*((1.5 + .8517*x)^2), where x is the category midpoint in mg at the peak of this conversion curve. when category midpoint was greater than 1150mg, we used this same PAEE value for higher accelerations. We then multiplied by 0.001*60 to express in units of kJ/kg/hour.
2. These are the average hours or energy expenditure spent in each category in present study.
3. This was calculated by multiplying the average hours per day spent in each category (column 4) by the energy expenditure (column 5).

FMVPAEE, fraction of moderate or vigorous physical activity energy expenditure; FLPAEE, fraction of light physical activity energy expenditure; FMPAEE, fraction of moderate physical activity energy expenditure; FVPAEE, fraction of vigorous physical activity energy expenditure.

Reference

[1] Strain T, Wijndaele K, Dempsey PC, et al. Wearable-device-measured physical activity and future health risk. Nat Med. 2020. 26(9): 1385-1391.

**Supplementary Table 2, Baseline Characteristics of included participants based on quantiles of FMVPE**

| **Quantiles of FMVPAEE(%)** | **1** | **2** | **3** | **4** | **P-value** |
| --- | --- | --- | --- | --- | --- |
| **N** | 23625 | 23626 | 23626 | 23625 |  |
| **PAEE(KJ/Kg/d)** | 33.76 ± 8.90 | 41.98 ± 7.83 | 48.56 ± 9.17 | 60.11 ± 13.36 | <0.01 |
| **FMVPAEE (%)** | 19.01 ± 4.33 | 27.78 ± 1.84 | 34 ± 2.02 | 42.05 ± 4.41 | <0.01 |
| **Age(years)** | 59.77 ± 6.81 | 56.86 ± 7.53 | 55.02 ± 7.70 | 52.97 ± 7.57 | <0.01 |
| **Sex, female (%)** | 14162 (59.94%) | 13673 (57.87%) | 12973 (54.91%) | 12397 (52.47%) | <0.01 |
| **Race, White (%)** | 21716 (91.92%) | 21595 (91.40%) | 21301 (90.16%) | 21146 (89.51%) | <0.01 |
| **Smoking status** | 12525 (53.02%) | 13412 (56.77%) | 13826 (58.52%) | 14084 (59.61%) | <0.01 |
| Never | 8958 (37.92%) | 8610 (36.44%) | 8241 (34.88%) | 8088 (34.23%) |  |
| Current | 2081 (8.81%) | 1536 (6.50%) | 1491 (6.31%) | 1396 (5.91%) |  |
| Quit | 56 (0.24%) | 57 (0.24%) | 55 (0.23%) | 43 (0.18%) |  |
| No answer | 5 (0.02%) | 11 (0.05%) | 13 (0.06%) | 14 (0.06%) |  |
| **Diet score** |  |  |  |  | 0.21 |
| 0 | 965 (4.08%) | 965 (4.08%) | 967 (4.09%) | 956 (4.05%) |  |
| 1 | 5291 (22.40%) | 5231 (22.14%) | 5278 (22.34%) | 5122 (21.68%) |  |
| 2 | 8809 (37.29%) | 8855 (37.48%) | 8755 (37.06%) | 8664 (36.67%) |  |
| 3 | 6366 (26.95%) | 6308 (26.70%) | 6383 (27.02%) | 6536 (27.67%) |  |
| 4 | 2194 (9.29%) | 2267 (9.60%) | 2243 (9.49%) | 2347 (9.93%) |  |
| **Alcohol consumption(times)** |  |  |  |  | <0.01 |
| Daily | 5383 (22.80%) | 5466 (23.15%) | 5419 (22.96%) | 5313 (22.51%) |  |
| 3-4/week | 5203 (22.04%) | 5955 (25.22%) | 6551 (27.75%) | 6830 (28.94%) |  |
| 1-2/week | 5468 (23.16%) | 6002 (25.42%) | 6044 (25.60%) | 6180 (26.19%) |  |
| 1-3/month | 2856 (12.10%) | 2621 (11.10%) | 2413 (10.22%) | 2381 (10.09%) |  |
| Occasionally | 2953 (12.51%) | 2234 (9.46%) | 2013 (8.53%) | 1790 (7.58%) |  |
| Never | 1747 (7.40%) | 1331 (5.64%) | 1165 (4.94%) | 1106 (4.69%) |  |
| **Mobility limitation (%)** | 1083 (4.58%) | 382 (1.62%) | 244 (1.03%) | 188 (0.80%) | <0.01 |
| **In employment (%)** | 11056 (46.80%) | 14218 (60.18%) | 15838 (67.04%) | 17164 (72.65%) | <0.01 |
| **WC (cm^2^)** | 92.51 ± 14.04 | 89.04 ± 12.79 | 87.01 ± 12.25 | 84.83 ± 11.78 | <0.01 |
| **BMI(Kg/m^2^)** | 28.26 ± 5.17 | 27.01 ± 4.47 | 26.17 ± 4.06 | 25.40 ± 3.78 | <0.01 |
| Normal weight (<25) | 6488 (27.46%) | 8470 (35.85%) | 10150 (42.96%) | 11986 (50.73%) |  |
| Overweight (>=25, <30) | 9843 (41.66%) | 10290 (43.55%) | 9900 (41.90%) | 9021 (38.18%) |  |
| Obese (>=30) | 7294 (30.87%) | 4866 (20.60%) | 3576 (15.14%) | 2618 (11.08%) |  |
| **TDI** | -1.70 ± 2.82 | -1.84 ± 2.76 | -1.75 ± 2.82 | -1.60 ± 2.87 | <0.01 |
| **DBP (mmHg)** | 82.55 ± 10.29 | 82.07 ± 10.32 | 81.25 ± 10.18 | 80.84 ± 10.21 | <0.01 |
| **SBP (mmHg)** | 141.89 ± 19.06 | 139.29 ± 18.73 | 137.35 ± 18.36 | 135.99 ± 18.16 | <0.01 |
| **Total [cholesterol](javascript:;)(mmol/L)** | 5.80 ± 1.11 | 5.79±1.07 | 5.77±1.04 | 5.77±1.06 |  |
| **Cholesterol medication** | 8.0 | 4.8 | 3.0 | 1.8 |  |

PAEE, physical activity energy expenditure; FMVPAEE, fraction of moderate or vigorous physical activity energy expenditure; TDI, Townsend indicator of deprivation; DBP, diastolic blood pressure; SBP, systolic blood pressure.

**Supplementary Table 3, Associations between PAEE and T2DM**

| **PAEE(median, KJ/Kg/d)** | **Model 1** | **Model 2** | **Model 3** |
| --- | --- | --- | --- |
| **Q1(32)** | Reference | Reference | Reference |
| **Q2(41)** | 0.56(0.49,0.62) | 0.84(0.75,0.95) | 0.85(0.76,0.96) |
| **Q3(49)** | 0.42(0.37,0.47) | 0.77(0.68,0.88) | 0.79(0.69,0.91) |
| **Q4(62)** | 0.25(0.22,0.29) | 0.62(0.52,0.72) | 0.63(0.54,0.75) |
| **P for trend** | <0.01 | <0.01 | <0.01 |
| **Per SD increase** | 0.54(0.51,0.57) | 0.82(0.77,0.87) | 0.83(0.78,0.98) |

Model 1: unadjusted

Model 2: age, sex, race, waist circumference, BMI, smoking status, alcohol intake frequency, diet score, employment status, TDI, mobility limitation.

Model 3: Model 2 in addition to SBP, DBP, cholesterol medication, history of myocardial infarction.

PAEE, physical activity energy expenditure; BMI, body mass index TDI, Townsend indicator of deprivation; DBP, diastolic blood pressure; SBP, systolic blood pressure.

**Supplementary Table 4, Associations Between VPAEE and risk of T2DM**

| **VPAEE(median, KJ/Kg/d)** | **Model 1** | **Model 2** | **Model 3** | **Model 3+PAEE** |
| --- | --- | --- | --- | --- |
| **Q1(0)** | Reference | Reference | Reference | Reference |
| **Q2(0.6)** | 0.74(0.67,0.83) | 0.99(0.88,1.11) | 1.00(0.89,1.12) | 1.04(0.92,1.17) |
| **Q3(1.8)** | 0.49(0.43,0.56) | 0.76(0.66,0.87) | 0.77(0.67,0.88) | 0.83(0.71,0.96) |
| **Q4(4.3)** | 0.26(0.22,0.31) | 0.52(0.44,0.62) | 0.53(0.45,0.63) | 0.60(0.49,0.740 |
| **P for trend** | <0.01 | <0.01 | <0.01 | <0.01 |
| **Per SD increase** | 0.43(0.38,0.48) | 0.68(0.61,0.75) | 0.68(0.62,0.76) | 0.73(0.64,0.82) |

Model 1: unadjusted

Model 2: age, sex, race, waist circumference, body mass index, smoking status, alcohol intake frequency, diet score, employment status, townsend deprivation index, mobility limitation.

Model 3: Model 2 in addition to SBP, DBP, cholesterol medication, history of myocardial infarction.

PAEE, physical activity energy expenditure; MVPA, moderate or vigorous physical activity; TDI, Townsend indicator of deprivation; DBP, diastolic blood pressure; SBP, systolic blood pressure; VPAEE, vigorous physical activity energy expenditure

**Supplementary Table 5, Associations Between MPAEE and risk of T2DM**

| **MPAEE(median, KJ/Kg/d)** | **Model 1** | **Model 2** | **Model 3** | **Model 3+PAEE** |
| --- | --- | --- | --- | --- |
| **Q1(6.7)** | Reference | Reference | Reference | Reference |
| **Q2(11.5)** | 0.57(0.51,0.64) | 0.86(0.77,0.97) | 0.88(0.78,0.99) | 0.96(0.84,1.10) |
| **Q3(16.3)** | 0.40(0.35,0.45) | 0.73(0.64,0.84) | 0.75(0.66,0.86) | 0.87(0.73,1.05) |
| **Q4(24.7)** | 0.26(0.23,0.31) | 0.61(0.52,0.72) | 0.63(0.54,0.74) | 0.81(0.63,1.05) |
| **P for trend** | <0.01 | <0.01 | <0.01 | <0.01 |
| **Per SD increase** | 0.53(0.50,0.57) | 0.79(0.75,0.84) | 0.81(0.76,0.86) | 0.82(0.73,0.93) |

Model 1: unadjusted

Model 2: age, sex, race, waist circumference, body mass index, smoking status, alcohol intake frequency, diet score, employment status, townsend deprivation index, mobility limitation.

Model 3: Model 2 in addition to SBP, DBP, cholesterol medication, history of myocardial infarction.

PAEE, physical activity energy expenditure; MPAEE, moderate physical activity energy expenditure; TDI, Townsend indicator of deprivation; DBP, diastolic blood pressure; SBP, systolic blood pressure.

**Supplementary Table 6, Associations Between LPAEE and risk of T2DM**

| **LPAEE(median, KJ/Kg/d)** | **Model 1** | **Model 2** | **Model 3** | **Model 3+PAEE** |
| --- | --- | --- | --- | --- |
| **Q1(24.1)** | Reference | Reference | Reference | Reference |
| **Q2(29.1)** | 0.60(0.53,0.68) | 0.87(0.77,0.98) | 0.87(0.77,0.98) | 1.13(0.98,1.30) |
| **Q3(33.0)** | 0.53(0.47,0.60) | 0.94(0.82,1.06) | 0.95(0.83,1.08) | 1.46(1.22,1.74) |
| **Q4(38.2)** | 0.35(0.30,0.40) | 0.76(0.66,0.88) | 0.78(0.67,0.90) | 1.56(1.22,1.99) |
| **P for trend** | <0.01 | <0.01 | <0.01 | <0.01 |
| **Per SD increase** | 0.65(0.62,0.68) | 0.91(0.87,0.96) | 0.92(0.87,0.97) | 1.44(1.28,1.62) |

Model 1: unadjusted

Model 2: age, sex, race, waist circumference, body mass index, smoking status, alcohol intake frequency, diet score, employment status, townsend deprivation index, mobility limitation.

Model 3: Model 2 in addition to SBP, DBP, cholesterol medication, history of myocardial infarction.

PAEE, physical activity energy expenditure; MVPA, moderate or vigorous physical activity; TDI, Townsend indicator of deprivation; DBP, diastolic blood pressure; SBP, systolic blood pressure; LPAEE, light physical activity energy expenditure.

**Supplementary Table 7, Associations between FMVPAEE and T2DM**

| **FMVPAEE(median, %)** | **Model 1** | **Model 2** | **Model 3** | **Model3+PAEE** |
| --- | --- | --- | --- | --- |
| **Q1(20.0)** | Reference | Reference | Reference | Reference |
| **Q2(27.8)** | 0.65(0.58,0.72) | 0.91(0.81,1.02) | 0.93(0.83,1.04) | 0.97(0.85,1.09) |
| **Q3(33.9)** | 0.42(0.37,0.48) | 0.72(0.63,0.83) | 0.75(0.65,0.86) | 0.80(0.68,0.93) |
| **Q4(41.4)** | 0.25(0.21,0.29) | 0.51(0.43,0.60) | 0.52(0.44,0.62) | 0.58(0.47,0.72) |
| **P for trend** | <0.01 | <0.01 | <0.01 | <0.01 |
| **Per 10% increase** | 0.56(0.53,0.59) | 0.78(0.73,0.82) | 0.79(0.74,0.83) | 0.81(0.74,0.87) |

Model 1: unadjusted

Model 2: age, sex, race, waist circumference,BMI , smoking status, alcohol intake frequency, diet score, employment status, townsend deprivation index, mobility limitation.

Model 3: Model 2 in addition to SBP, DBP, cholesterol medication, history of myocardial infarction.

FMVPAEE, moderate or vigorous physical activity energy expenditure; PAEE, physical activity energy expenditure; BMI, body mass index; TDI, Townsend indicator of deprivation; DBP, diastolic blood pressure; SBP, systolic blood pressure.

**Supplementary Table 8, Associations Between FVPAEE and risk of T2DM**

| **FVPAEE (median, %)** | **Model 1** | **Model 2** | **Model 3** | **Model3+PAEE** |
| --- | --- | --- | --- | --- |
| **Q1(18.2)** | Reference | Reference | Reference | Reference |
| **Q2(24.5)** | 0.91(0.81,1.01) | 1.03(0.92,1.16) | 1.03(0.92,1.16) | 1.06(0.94,1.18) |
| **Q3(29.3)** | 0.55(0.48,0.62) | 0.76(0.66,0.87) | 0.77(0.67,0.88) | 0.82(0.71,0.94) |
| **Q4(35.6)** | 0.33(0.28,0.39) | 0.56(0.48,0.66) | 0.57(0.48,0.67) | 0.64(0.53,0.77) |
| **P for trend** | <0.01 | <0.01 | <0.01 | <0.01 |
| **Per SD increase** | 0.55(0.51,0.59) | 0.74(0.69,0.80) | 0.75(0.69,0.81) | 0.79(0.73,0.86) |

Model 1: unadjusted

Model 2: age, sex, race, waist circumference, body mass index, smoking status, alcohol intake frequency, diet score, employment status, townsend deprivation index, mobility limitation.

Model 3: Model 2 in addition to SBP, DBP, cholesterol medication, history of myocardial infarction.

PAEE, physical activity energy expenditure; MVPA, moderate or vigorous physical activity; TDI, Townsend indicator of deprivation; DBP, diastolic blood pressure; SBP, systolic blood pressure; FVPE, fraction of vigorous physical energy .

**Supplementary Table 9, Associations Between FMPAEE and risk of T2DM**

| **FMPAE E(median, %)** | **Model 1** | **Model 2** | **Model 3** | **Model3+PAEE** |
| --- | --- | --- | --- | --- |
| **Q1(18.2)** | Reference | Reference | Reference | Reference |
| **Q2(24.5)** | 0.65(0.58,0.73) | 0.92(0.81,1.03) | 0.94(0.83,1.05) | 1.01(0.89,1.14) |
| **Q3(29.3)** | 0.48(0.42,0.54) | 0.79(0.69,0.90) | 0.82(0.71,0.93) | 0.92(0.80,1.07) |
| **Q4(35.6)** | 0.32(0.28,0.38) | 0.64(0.55,0.75) | 0.66(0.57,0.77) | 0.80(0.67,0.96) |
| **P for trend** | <0.01 | <0.01 | <0.01 | <0.01 |
| **Per SD increase** | 0.53(0.50,0.57) | 0.79(0.75,0.84) | 0.81(0.76,0.86) | 0.82(0.73,0.93) |

Model 1: unadjusted

Model 2: age, sex, race, waist circumference, body mass index, smoking status, alcohol intake frequency, diet score, employment status, townsend deprivation index, mobility limitation.

Model 3: Model 2 in addition to SBP, DBP, cholesterol medication, history of myocardial infarction.

PAEE, physical activity energy expenditure; MVPA, moderate or vigorous physical activity; TDI, Townsend indicator of deprivation; DBP, diastolic blood pressure; SBP, systolic blood pressure; FMPE, fraction of moderate physical energy

**Supplementary Table 10, Associations Between FLPAEE and risk of T2DM**

| **FLPAEE(median, %)** | **Model 1** | **Model** | **Model 3** | **Model3+PAEE** |
| --- | --- | --- | --- | --- |
| **Q1(58.6)** | Reference | Reference | Reference | Reference |
| **Q2(66.1)** | 1.72(1.44,2.06) | 1.42(1.18,1.69) | 1.43(1.20,1.71) | 1.36(1.13,1.64) |
| **Q3(72.2)** | 2.63(2.22,3.10) | 1.78(1.51,2.11) | 1.78(1.50,2.11) | 1.65(1.37,2.00) |
| **Q4(80.0)** | 4.07(3.47,4.77) | 1.96(1.65,2.32) | 1.91(1.61,2.27) | 1.71(1.38,2.12) |
| **P for trend** | <0.01 | <0.01 | <0.01 | <0.01 |
| **Per 10% increase** | 1.70(1.63,1.79) | 1.26(1.20,1.33) | 1.24(1.18,1.31) | 1.22(1.13,1.31) |

Model 1: unadjusted

Model 2: age, sex, race, waist circumference, body mass index, smoking status, alcohol intake frequency, diet score, employment status, townsend deprivation index, mobility limitation.

Model 3: Model 2 in addition to SBP, DBP, cholesterol medication, history of myocardial infarction.

PAEE, physical activity energy expenditure; MVPA, moderate or vigorous physical activity; TDI, Townsend indicator of deprivation; DBP, diastolic blood pressure; SBP, systolic blood pressure; FMPE, fraction of light physical energy

**Supplementary Table 11, Associations between joint distribution of PAEE and FMVPAEE and T2DM**

| **PAEE(median, KJ/Kg/d)** | **FMVPAEE (median,%)** | **Model 1** | **Model 2** | **Model 3** |
| --- | --- | --- | --- | --- |
| **Q1(32)** | Q1(20) | Reference | Reference | Reference |
|  | Q2(28) | 0.76(0.64,0.90) | 0.95(0.80,1.13) | 0.98(0.82,1.16) |
|  | Q3(34) | 0.56(0.41,0.75) | 0.75(0.55,1.03) | 0.77(0.56,1.05) |
|  | Q4(41) | NA | NA | NA |
| **Q2(41)** | Q1(20) | 0.61(0.51,0.73) | 0.93(0.78,1.11) | 0.94(0.79,1.13) |
|  | Q2(28) | 0.52(0.44,0.61) | 0.84(0.71,1.00) | 0.86(0.73,1.02) |
|  | Q3(34) | 0.40(0.32,0.50) | 0.70(0.56,0.88) | 0.73(0.58,0.91) |
|  | Q4(41) | NA | NA | NA |
| **Q3(49)** | Q1(20) | NA | NA | NA |
|  | Q2(28) | 0.43(0.36,0.53) | 0.84(0.69,1.04) | 0.88(0.72,1.08) |
|  | Q3(34) | 0.33(0.27,0.40) | 0.68(0.55,0.83) | 0.71(0.58,0.86) |
|  | Q4(41) | 0.32(0.25,0.41) | 0.63(0.49,0.82) | 0.65(0.50,0.840 |
| **Q4(62)** | Q1(20) | NA | NA | NA |
|  | Q2(28) | NA | NA | NA |
|  | Q3(34) | 0.31(0.24,0.39) | 0.73(0.57,0.94) | 0.76(0.59,0.98) |
|  | Q4(41) | 0.16(0.13,0.20) | 0.44(0.35,0.55) | 0.46(0.37,0.57) |

Model 1: unadjusted

Model 2: age, sex, race, waist circumference, BMI, smoking status, alcohol intake frequency, diet score, employment status, TDI, mobility limitation.

Model 3: Model 2 in addition to SBP, DBP, cholesterol medication, history of myocardial infarction.

PAEE, physical activity energy expenditure; FMVPAEE, fraction of moderate or vigorous physical activity energy expenditure; TDI, Townsend indicator of deprivation; BMI, body mass index; DBP, diastolic blood pressure; SBP, systolic blood pressure.

NA means limited participants in these groups

**Supplementary Figure 1, Timeline of recruitment, data collection and follow-up for included participants**


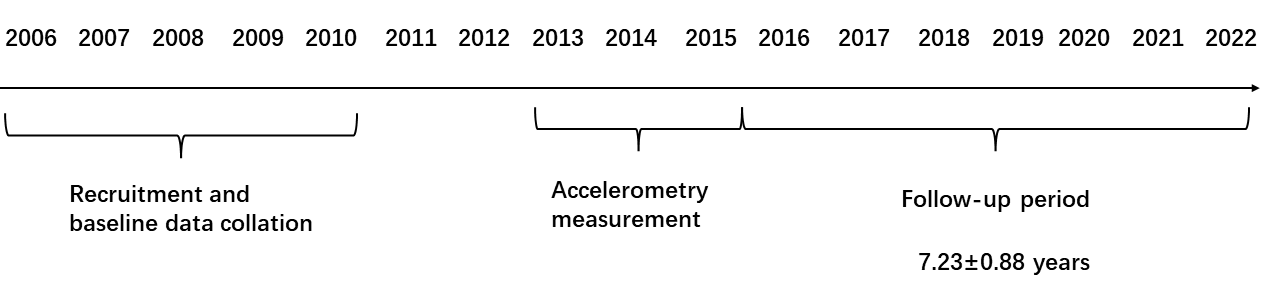


**Supplementary Figure 2. Flow diagram of participants in the study**


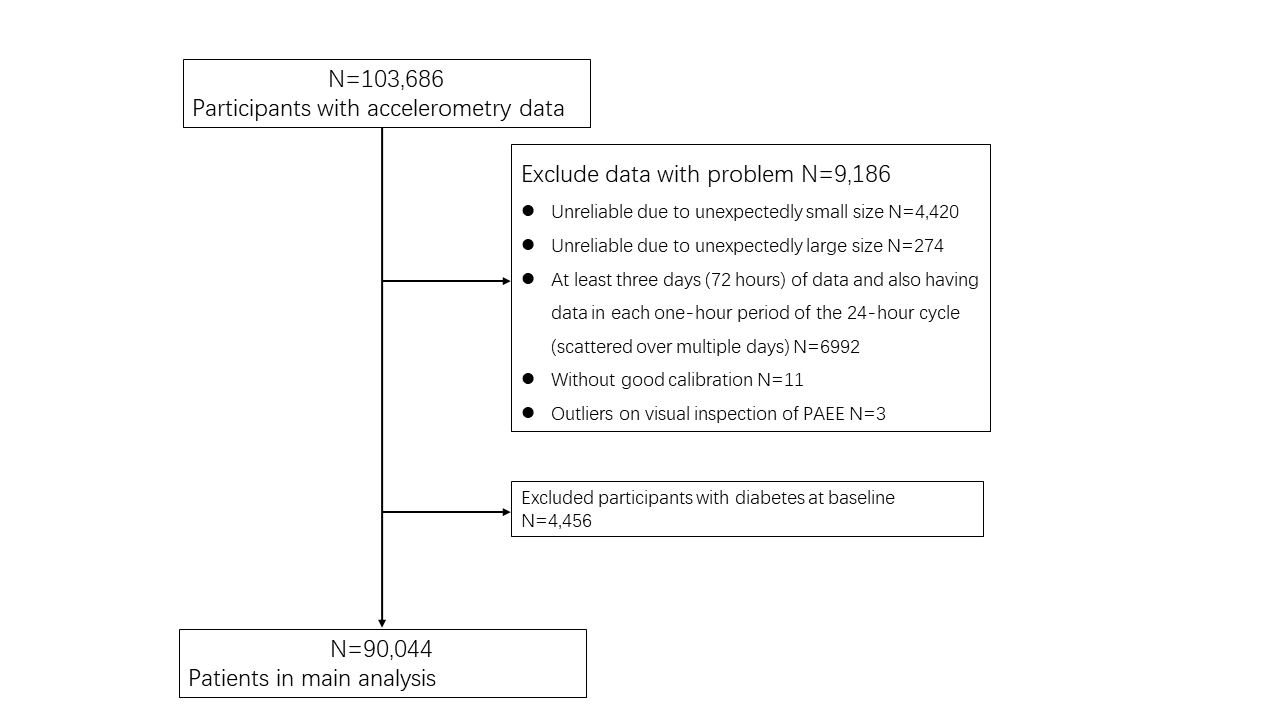


**Supplementary Figure 3, Subgroup analysis of the association between PAEE and FMVPAEE and T2DM**


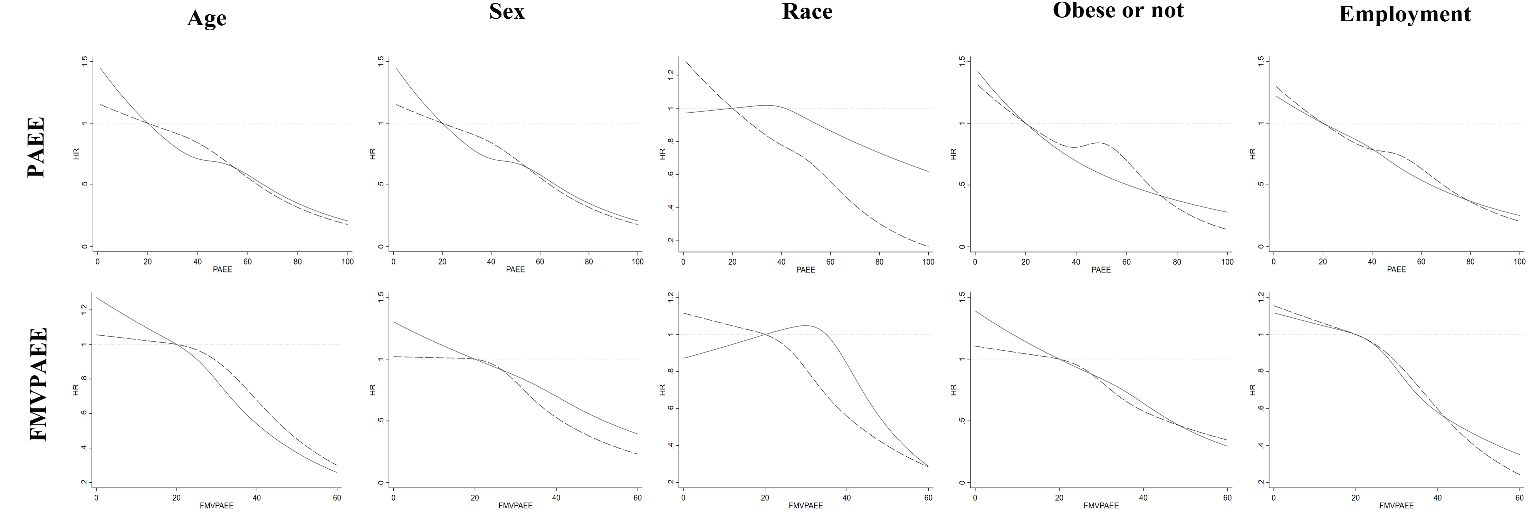

Supplement: Supplementary file 1 [file DataSheet_1.docx]
